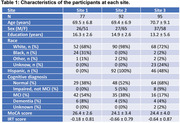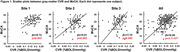# Cerebrovascular reactivity (CVR) MRI as a biomarker for cerebral small vessel disease (SVD) related cognitive decline: Multi‐site validation in the MarkVCID Consortium

**DOI:** 10.1002/alz.093817

**Published:** 2025-01-09

**Authors:** Peiying Liu, Zixuan Lin, Kaisha Hazel, George Pottanat, Cuimei Xu, Dengrong Jiang, Jay J Pillai, Emma Lucke, Christopher E. Bauer, Brian T Gold, Steven M. Greenberg, Karl G Helmer, Kay Jann, Gregory A Jicha, Joel H. Kramer, Pauline Maillard, Rachel M. Mulavelil, Claudia L Satizabal, Kristin Schwab, Sudha Seshadri, Herpreet Singh, Angel G Velarde, Danny JJ Wang, Rita Kalyani, Abhay Moghekar, Paul B. Rosenberg, Sevil Yasar, Marilyn S. Albert, Hanzhang Lu

**Affiliations:** ^1^ University of Maryland School of Medicine, Baltimore, MD USA; ^2^ Johns Hopkins University School of Medicine, Baltimore, MD USA; ^3^ University of Kentucky, Lexington, KY USA; ^4^ Massachusetts General Hospital, Harvard Medical School, Boston, MA USA; ^5^ Massachusetts General Hospital, Boston, MA USA; ^6^ University of Southern California, Los Angeles, CA USA; ^7^ University of California San Francisco, San Francisco, CA USA; ^8^ Alzheimer’s Disease Research Center, University of California Davis, Sacramento, CA USA; ^9^ Glenn Biggs Institute for Alzheimer’s & Neurodegenerative Diseases, University of Texas Health Science Center, San Antonio, TX USA; ^10^ Glenn Biggs Institute for Alzheimer’s & Neurodegenerative Diseases, University of Texas Health Sciences Center at San Antonio, San Antonio, TX USA; ^11^ Glenn Biggs Institute for Alzheimer’s & Neurodegenerative Diseases, University of Texas Health San Antonio, San Antonio, TX USA; ^12^ Johns Hopkins University, Baltimore, MD USA

## Abstract

**Background:**

Cerebral small vessel disease (SVD) related vascular contributions represent a major factor contributing to cognitive decline and dementia (VCID) in older adults. However, there has not been a validated biomarker for the diagnosis and treatment monitoring of this condition. Recently, the US National Institute on Neurological Disorders and Stroke (NINDS) funded the MarkVCID Consortium to identify and validate clinical‐trial‐ready biomarkers for VCID. Cerebrovascular reactivity (CVR) MRI is one of the selected biomarkers that underwent multi‐site testing in the Consortium. The present study aimed to report the relationship between CVR and cognitive function at independent sites, based on a pre‐specified hypothesis.

**Method:**

CVR, the ability of cerebral small vessels to dilate upon stimulus, is thought to directly reflect physiological function of the brain microvasculature. Based on previous single‐site findings, the pre‐defined hypothesis was that CVR will be associated with the global cognitive function measured by Montreal Cognitive Assessment (MoCA) after adjusting for age, sex, and education, and this association will be observed in data collected and analyzed at each individual site. A total of 264 older participants from three sites were included (Table 1). Each site performed an identical CVR MRI procedure using 5% CO2 inhalation, and a standardized clinical and cognitive evaluation. Multi‐linear regression analysis was conducted on a site‐by‐site basis to examine the pre‐defined hypothesis.

**Result:**

Gray‐matter CVR showed a positive association with MoCA score after adjustment for age, sex, and education, in which participants with higher CVR had higher MoCA scores. This relationship was reproduced at each site (Fig.1, p<0.05 for each), confirming our pre‐specified hypothesis. In the secondary analysis of all data together, higher gray‐matter CVR was found to be significantly associated with better executive function measure of item response theory (IRT) score (ß = 2.87, p = 0.003).

**Conclusion:**

The present study evaluated the relationship between CVR and cognition in a multi‐center setting. CVR was found to be positively associated with global cognitive function measured by the MoCA, which was shown to be reproducible across different sites with diverse cohorts. These findings support the utility of CVR as a biomarker in future clinical trials of SVD and VCID.